# Teaching Python with team‐based learning: using cloud‐based notebooks for interactive coding education

**DOI:** 10.1002/2211-5463.70097

**Published:** 2025-08-11

**Authors:** Nuno S. Osório, Leonardo D. Garma

**Affiliations:** ^1^ Life and Health Sciences Research Institute (ICVS), School of Medicine University of Minho Braga Portugal; ^2^ ICVS/3B's‐PT Government Associate Laboratory Braga Portugal; ^3^ Breast Cancer Clinical Research Unit Centro Nacional de Investigaciones Oncológicas – CNIO Madrid Spain

**Keywords:** bioinformatics education, biomedical students, interactive notebooks, Python instruction, student engagement

## Abstract

Computer programming and bioinformatics are increasingly essential topics in life sciences research, facilitating the analysis of large and complex ‘omics’ datasets. However, they remain challenging for students without a background in mathematics or computing. To address challenges in teaching programming within biomedical education, this study integrates team‐based learning (TBL) with cloud‐hosted interactive Python notebooks, targeting enhanced student engagement, understanding, and collaboration in bioinformatics in two Masters level classes with 28 biomedical students in total. Four interactive notebooks covering Python basics and practical bioinformatics applications—ranging from data manipulation to multi‐omics analysis—were developed. Hosted on github and integrated with Google Colaboratory, these notebooks ensured equal access and eliminated technical barriers for students with varied computing setups. During the TBL session, students were highly engaged with the notebooks, which led to a greater interest in Python and increased confidence in using bioinformatics tools. Feedback highlighted the value of TBL and interactive notebooks in enriching the learning experience, while also identifying a need for further development in bioinformatics research skills. Although more validity evidence is needed in future studies, this blended, cloud‐based TBL approach effectively made bioinformatics education more accessible and engaging, suggesting its potential for enhancing computational training across life sciences.

## Graphical abstract

This study presents a novel approach to teaching Python and bioinformatics using team‐based learning and cloud‐hosted notebooks. By integrating interactive coding into biomedical education, the method improves accessibility, student engagement, and confidence—especially for those without a computing background.

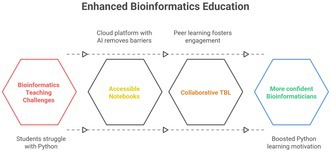

**Abbreviations**
IRA: Individual Readiness Assurance
MCQ: multiple‐choice question
TBL: Team‐Based Learning
TRA: Team Readiness Assurance

In an increasingly data‐driven life sciences landscape, the integration of computational tools into biomedical education is essential. The widespread adoption of technologies such as next‐generation sequencing (NGS) and mass spectrometry has generated vast ‘omics’ datasets, which require specialized computational tools and skills for effective analysis [1]. However, a pressing issue has emerged: teaching Python—a central language in bioinformatics and data science [2]—to students with limited programming backgrounds remains a significant pedagogical challenge in biomedical education. Current teaching approaches often do not address students' unique needs in computational thinking and engagement with complex biological data [3, 4, 5].

Despite the rise of computer‐based and online learning, disengagement persists, especially in asynchronous and self‐paced settings, where students frequently experience reduced motivation and isolation [6, 7, 8, 9].

These challenges can lead to lower learning outcomes and decreased course completion rates [10, 11]. Addressing this issue necessitates pedagogical strategies that actively foster student engagement, a key predictor of academic success and persistence [12, 13]. Engagement is especially critical in disciplines such as programming, where students benefit from interactive, applied learning and peer collaboration.

Team‐based learning (TBL), grounded in constructivist theories, has shown promise as a method to deepen learning, enhance engagement, and build teamwork skills in health sciences education [14, 15, 16, 17, 18, 19, 20, 21]. In TBL, students work in permanent teams throughout the course, engaging in readiness assurance processes and application‐focused exercises that demand active participation and peer teaching [19]. This collaborative structure is particularly effective in counteracting the isolation and passivity common in online and large‐enrollment courses, including programming education. Furthermore, research indicates that TBL not only supports deeper conceptual understanding but also enhances engagement through social interaction, shared goals, and immediate feedback [22, 23]. These elements are essential for fostering a learning environment where students feel connected, supported, and invested in their learning process, factors that are consistently linked to higher academic performance and persistence [24].

While previous studies have demonstrated the applicability of team‐based learning (TBL) in data science and systems programming contexts [25, 26], there remains a gap in the literature regarding its use in bioinformatics. To date, no research has specifically examined the integration of TBL with computational tools like Python notebooks to support learning in this domain.

This study aims to bridge this gap by combining TBL with cloud‐based interactive Python notebooks, creating a novel framework for teaching Python programming in biomedical education. Cloud‐based interactive notebooks like jupyter allow students to write, execute, and visualize code in real time, fostering active learning and bridging theory with application [27, 28, 29]. Integrating these notebooks in cloud environments reduces technical barriers, enabling students to focus on learning rather than software management [30]. Recent applications of these cloud‐based environments, particularly in gene expression, proteomics, and biomarker discovery, underscore their utility in bioinformatics education [31, 32, 33]. This study thus proposes an innovative, hybrid approach, hypothesizing that combining TBL with cloud‐based coding environments will significantly enhance student engagement, comprehension, and collaboration in Python programming for bioinformatics.

By framing this study as a response to current pedagogical challenges in biomedical programming education, it contributes to ongoing discussions about how best to prepare biomedical students for data‐centric careers in life sciences. Through this blended approach, we aimed to provide insights that advance both Python programming pedagogy and the broader field of bioinformatics education, where effective computational training is essential for future scientific discovery.

We implemented an innovative approach combining team‐based learning (TBL) with cloud‐hosted interactive Python notebooks, aimed at enhancing engagement, conceptual understanding, and access to programming tools. The results indicate that this integrated approach successfully fostered active learning and critical thinking, with students reporting increased motivation, deeper comprehension of Python programming concepts, and greater appreciation of its relevance in bioinformatics. The TBL framework encouraged collaboration and peer‐supported learning, while the use of interactive notebooks ensured equitable access and reduced technical friction. Students showed a marked increase in interest and confidence in using Python, underscoring the potential of this pedagogical model. These findings suggest that the combination of TBL and cloud‐based tools offers a promising strategy for computational education in the biomedical sciences, and they provide a foundation for further research and course development in this evolving field.

## Materials and methods

### Methodology

This study explored the design and execution of innovative pedagogical approaches through a dedicated TBL session across two postgraduate courses at the School of Medicine, University of Minho, Portugal, designed for students with limited programming experience. The first course, Bioinformatics in Health Sciences, included sixteen participants (*N* = 16, 12 female, 4 male), predominantly master's and doctoral students with backgrounds in biochemistry, biology, or biomedicine, enrolled in this course. The second course, Single‐Cell Genomics for Beginners, introduced foundational single‐cell RNA sequencing techniques to 12 postgraduate students (*N* = 12, 10 female, 2 male) with backgrounds in biochemistry, biology, or biomedicine, similar to the first course.

### Team‐based learning (TBL)

TBL was adopted as the primary instructional strategy to foster collaboration and deepen learning, in line with its established benefits in active learning environments [18, 21]. The implementation followed the core phases of TBL: preparation, where students engaged with assigned materials independently; readiness assurance, which included individual and team assessments to reinforce accountability and understanding; and application, where teams worked collaboratively on problem‐solving tasks designed to promote critical thinking and the application of Python in bioinformatics contexts.

#### Preparation

Pre‐class preparation was grounded in the principles of flipped learning, where students reviewed foundational materials before class to maximize in‐class interactive learning time [34]. Resources included overviews of Anaconda [35], jupyter notebooks [36], and Google Colaboratory [37], which are essential platforms for executing and sharing Python code [36, 37, 38]. Additionally, students received a brief video introduction to the Team‐Based Learning (TBL) methodology. The core pre‐class material was an interactive jupyter notebook, hosted on github [39] and linked to Google Colaboratory, designed to provide a hands‐on introduction to Python programming. This setup enabled students to interact with code from any device without requiring local installations, making it highly accessible. Other platforms [40], bitbucket [41], sourceforge [42], launchpad [43], Figshare [44] and coding environments (Kaggle [45], cocalc [46], Paperspace [47]) were considered, but github and Google Colaboratory were chosen for their intuitive interfaces, capabilities to support collaborative coding environments, and widespread adoption [48].

Students were tasked with reviewing the pre‐class materials independently before the session and were divided into teams of four.

#### Readiness assurance

At the start of the TBL session, students completed a 30‐min Individual Readiness Assurance Test (IRA), consisting of 22 multiple‐choice questions on Python fundamentals (Supplementary Data S1) individually. The same test was administered as the team readiness assurance test (TRA), which lasted for 1 h and 30 min. During the TRA, students collaborated to reach consensus answers while using interactive Python notebooks, online resources, and supplemental materials to support their reasoning. Immediate feedback was provided in several ways: instructors circulated among teams to observe discussions and offer guidance; teams used the interactive notebooks to test code and validate their assumptions. This collaborative testing format, a core TBL component, is designed to improve student engagement and retention of content [19]. In our adaptation, allowing teams access to Python interactive notebooks and other resources during the TRA aimed to further leverage this collaborative phase for deeper learning and immediate application. Following the TRA, a 15‐min clarification session was conducted, where the instructor provided targeted feedback on areas of difficulty identified during the assessments. While we did not use IF‐AT cards or automated quiz tools that provide instant grading, the live use of code notebooks served as a meaningful and context‐relevant feedback mechanism, particularly for programming‐related questions.

#### Application

The TRA clarification session was followed by a 1‐h application case session, in which students worked through real‐world bioinformatics problems using additional interactive notebooks. These interactive notebooks, hosted on github and accessible via Google Colaboratory, allowed students to execute code, visualize data, and experiment with Python syntax in real time. Three interactive notebooks were developed: one focused on Python language fundamentals, and two on applying Python to bioinformatics case studies.

An additional self‐study interactive notebook was provided as post‐session material for further practice. While not part of the traditional TBL framework, this supplementary resource represented an instructional innovation aimed at supporting continued learning beyond structured sessions. Its inclusion offers a valuable point of reflection in student feedback, particularly regarding its perceived usefulness and role in reinforcing Python skills independently.

### Student feedback

To collect student ratings of teaching effectiveness, an optional and anonymous post‐course survey was administered through microsoft forms. The survey included seven items, adapted from the item bank of the Office of Institutional Research and Assessment (OIRA), Syracuse University, Syracuse, NY, USA and used a 5‐point Likert scale to assess students' perceptions of the course [49, 50]. Items included statements such as ‘My interest in Python has increased’, ‘The use of Team‐Based Learning (TBL) enriched my learning experience in this class’, and ‘Interactive notebooks were a valuable part of this course’. Survey data were analyzed and visualized using Python. In addition to formal survey responses, informal feedback was collected throughout the course, enabling real‐time fine adjustments to instructional practices.

### Data analysis and resources

Survey data were analyzed using Python, employing descriptive statistics to highlight trends. Visualizations were generated using seaborn and matplotlib. A co‐occurrence map of TBL multiple‐choice questions was created with vosviewer [51].

All course materials, including interactive Python notebooks, including the real‐world application exercises, and supplementary resources, are publicly accessible on github (https://github.com/Leo‐GG/Innovative‐Pedagogy‐in‐Bioinformatics), promoting continuous learning and reproducibility of this study's methodology.

### Ethical considerations

This study was conducted as an evaluation of pedagogical approaches within routine educational practice in postgraduate bioinformatics courses. The student feedback survey was optional, and data were collected entirely anonymously, with no personal identifiers recorded, ensuring participant confidentiality. Informed consent for participation in the survey was implied by its voluntary completion.

## Results

### Interactive notebooks structure and content

The design of the interactive notebooks aimed to provide comprehensive yet accessible content, striking a balance between covering a broad range of topics and presenting information clearly. Using this platform, students could execute, modify, and experiment with provided Python code blocks.

Four notebooks were prepared for the TBL implementation: an introductory Python notebook for pre‐class material and use during the TRA, two additional notebooks illustrating real‐world applications of Python for bioinformatics analysis, provided during the final application case session, and a self‐study notebook with exercises based on a practical example which was provided as an additional resource to promote continued learning beyond the classroom.

**Fig. 1:**
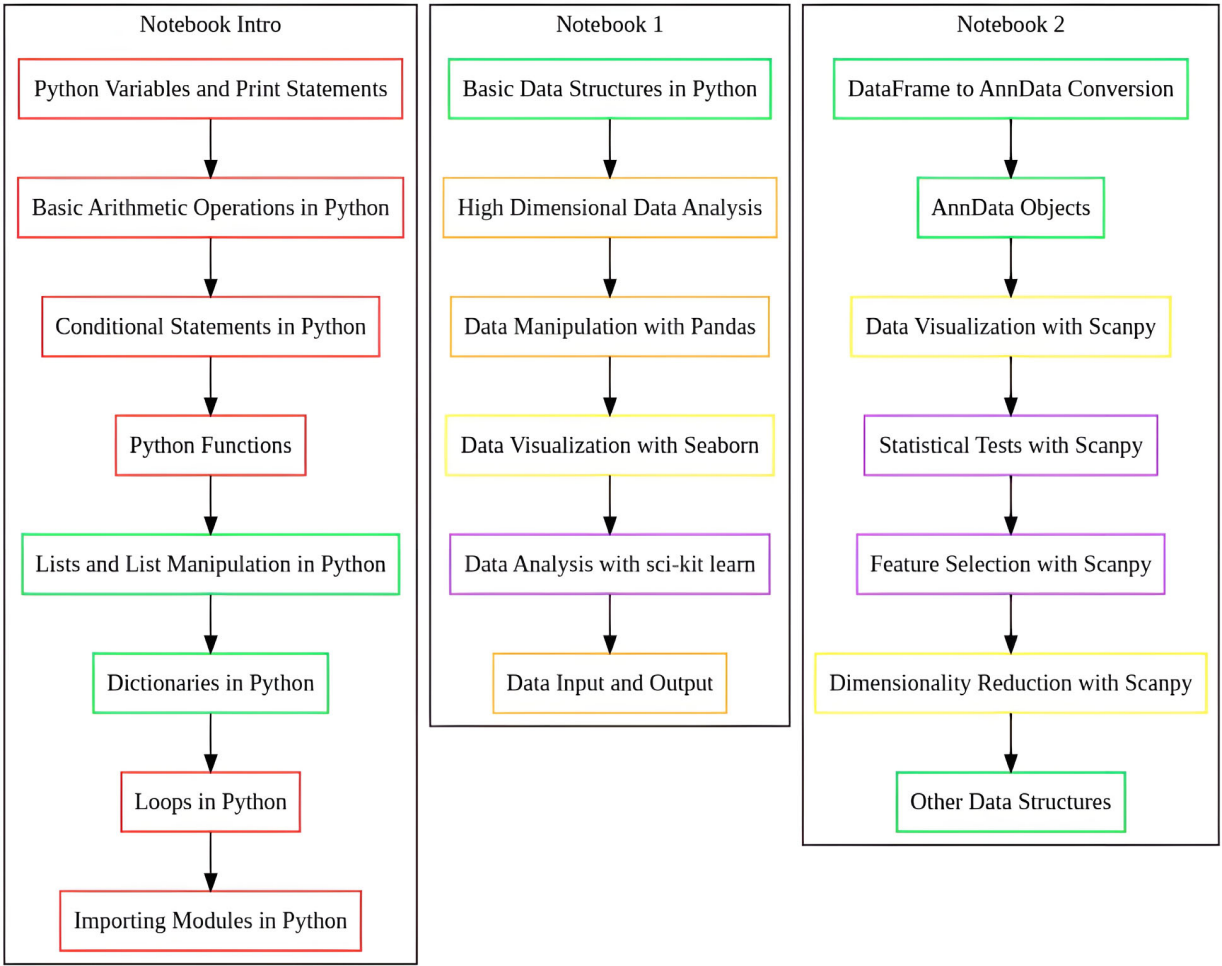
Content flow and interactions among three Python notebooks designed for the class. Each notebook focuses on different aspects of Python and data science, with shared colors indicating interconnected topics. The color legend is as follows: Red: Python Programming; Green: Data Structures; Orange: Data Manipulation; Yellow: Data Visualization; Purple: Data Analysis.

The notebooks were designed to provide a comprehensive introduction to Python for bioinformatics, from basic programming concepts to real‐world applications, ensuring the content was both wide‐ranging and understandable (Fig. 1). The content presented conceptually as ‘Notebook Intro’ in Fig. 1 (covering Python fundamentals) was delivered through the Introduction_to_Python.ipynb file, which served as pre‐class material and was also available as an in‐class resource during the Team Readiness Assurance test. The subsequent stages shown in Fig. 1 as ‘Notebook 1’ (focusing on data manipulation with pandas and other data analysis tasks) and ‘Notebook 2’ (covering bioinformatics analysis with tools like AnnData, Scanpy, and Muon) corresponded to two separate application notebooks (NB1_Intro_pandas.ipynb and NB2_Intro_scanpy.ipynb respectively) used during the final application case session. Collectively, these instructional notebooks covered Python fundamentals, data structures, and libraries and data structures applied in data analysis and bioinformatics (e.g., pandas [52], AnnData [53], Scanpy [54], Muon [55]) and demonstrated their application in real‐world bioinformatics scenarios.

The learning objectives across these three conceptual stages (Notebook Intro, Notebook 1, and Notebook 2 as depicted in Fig. 1) were structured to support a gradual development of programming and bioinformatics competencies. The ‘Notebook Intro’ stage aimed to establish core Python skills, the ‘Notebook 1’ stage focused on applying these skills to data analysis tasks, and the ‘Notebook 2’ stage extended these objectives to specific bioinformatics applications.

### Implementation and impact of cloud‐hosted interactive Python notebooks

Interactive Python notebooks have emerged as a powerful tool in bioinformatics education, promoting hands‐on learning and real‐time engagement. Notebooks allow students to easily execute, modify, and experiment with code. However, their integration into the classroom often faces technical challenges, ranging from hardware limitations to software conflicts [56]. These issues can create uncertainty and discourage the use of live coding in teaching sessions [57, 58].

**Fig. 2:**
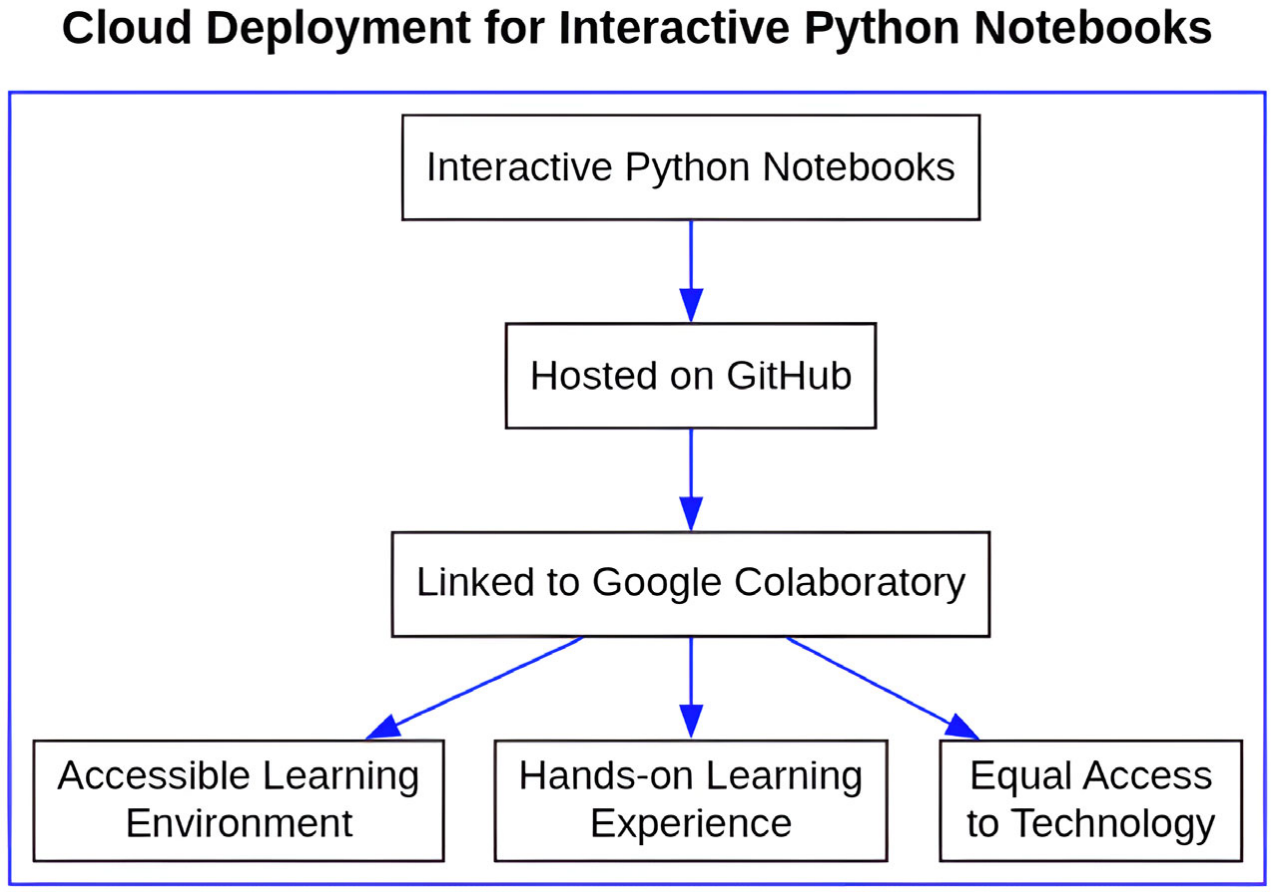
Selected Cloud Deployment Workflow for Interactive Python Notebooks in this Study. This flowchart illustrates the cloud‐based deployment process utilized for the interactive Python notebooks in our bioinformatics education approach. Notebooks were initially hosted on github for version control and accessibility. They were then linked to Google Colaboratory, a key step chosen to provide an accessible learning environment, facilitate a direct hands‐on coding experience for students without local setup barriers, and ensure equal access to necessary computational technology.

To address these challenges, we hosted the course's interactive Python notebooks on github and linked them directly to Google Colaboratory. This approach created a seamless and accessible learning environment, free from the technical difficulties associated with local computer environments. The cloud‐based platforms, freely available for academic and research purposes, facilitated an interactive learning experience that deepened students' understanding of Python and its applications in bioinformatics (Fig. 2).

The instructors observed that students engaged successfully with the notebooks both at home and in the classroom, executing the provided code and experimenting with modifications. This hands‐on approach reinforced the concepts presented in the notebooks. Furthermore, the cloud‐based approach ensured that all students, regardless of the hardware or operating system they had available, could access, read, and run the code without any difficulties.

This strategy not only eliminated potential technical obstacles but also promoted equal access to computational resources among students. It extended the learning environment beyond the physical classroom, enabling students to access the jupyter notebooks on any device. The implementation was highly successful, with no technical difficulties observed by the instructors nor reported by the students, indicating the robustness and reliability of this approach. Informal feedback from the students and formal evaluation (see the section ‘Students' Feedback’ below) indicated that the structure and content of the notebooks were well‐received, demonstrating the effectiveness of this method in conveying complex bioinformatics concepts and techniques.

We concluded that the use of cloud‐hosted interactive Python notebooks for bioinformatics instruction was a successful strategy. It provided a comprehensive, hands‐on, and accessible learning experience, enhancing students' understanding of Python and its real‐world applications in bioinformatics, and promoting equal access to technology.

### Implementation of team‐based learning

In preparation for the team‐based learning (TBL) session, students were given access to various resources 48 h in advance. These included slides and links introducing Python, its relevance in bioinformatics, and Google Colaboratory. The Python introductory notebook was made accessible, and students also received an explanation of TBL and guidance on accessing all pre‐class materials.

**Fig. 3:**
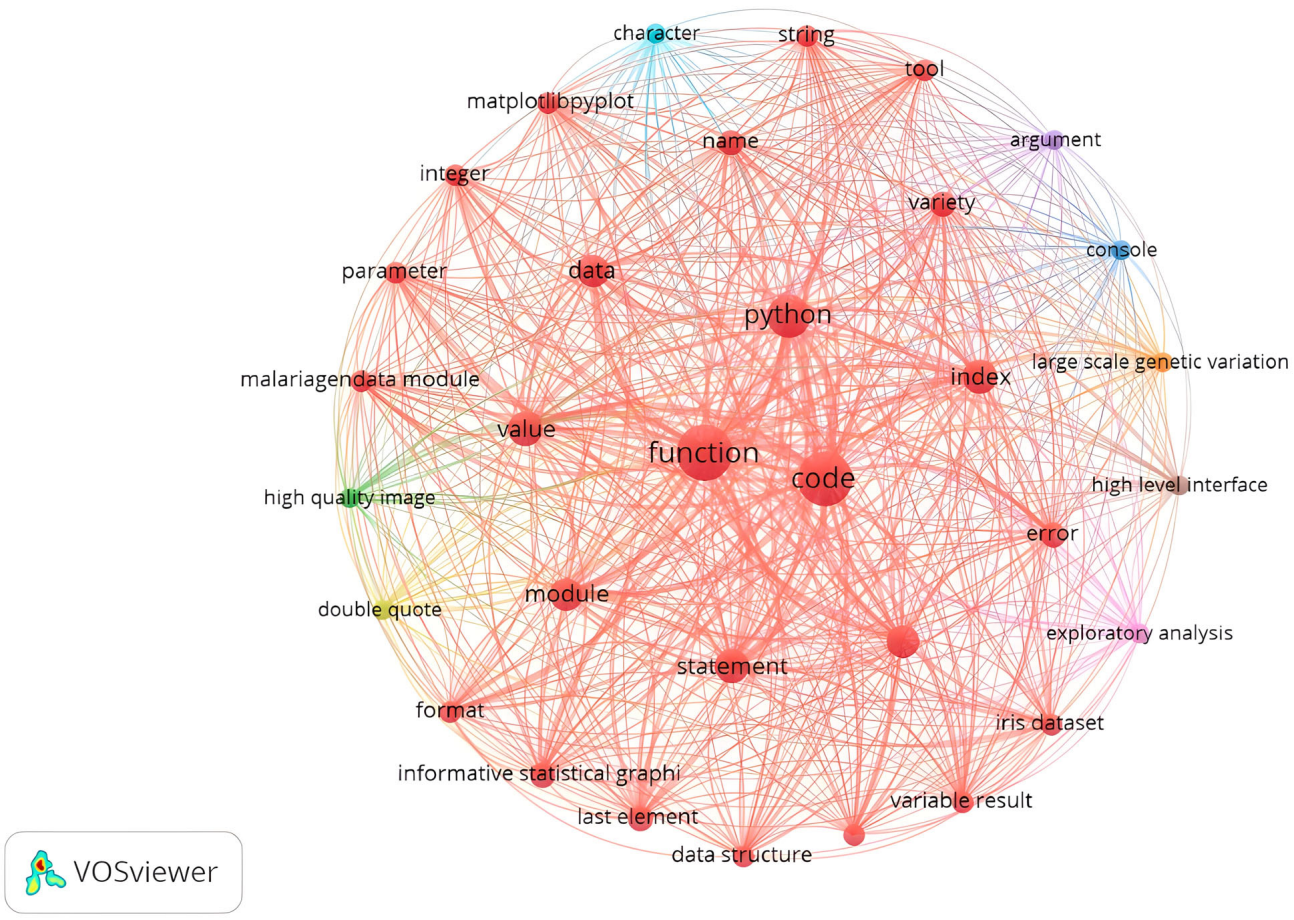
Co‐occurrence Map of MCQ Questions used in TBL. This figure presents a co‐occurrence map generated by the vosviewer software, based on the text analysis of the 22 questions used in the team‐based learning (TBL) strategy. The map visualizes the relationships among key terms extracted from the questions. Nodes: Each node represents a distinct term identified in the text analysis. The size of a node corresponds to the term's frequency in the analyzed text. Lines: The lines connecting the nodes represent the co‐occurrence of terms within the text. This co‐occurrence map provides a visual representation of the interconnectedness of the key concepts within the TBL strategy questions, offering insights into the underlying themes and patterns.

On the day of the activity, TBL served as the primary instructional strategy. Divided into groups, the students completed the IRA and TRA (see Methods section above) tests with 22 multiple‐choice questions (MCQ) on Python. We drew a co‐occurrence map of the MCQs to illustrate the relationships between key terms found in the questions, highlighting their prominence and conceptual connections (Fig. 3). Larger nodes such as function, code, and Python indicate that these terms appeared most frequently, suggesting a strong focus on fundamental programming constructs in the TBL materials. Other prominently connected terms include data, module, statement, and value, reflecting an emphasis on data handling, control structures, and modular code development. The density and interconnectedness of the map reveal the integrated nature of Python programming concepts taught through TBL. Clusters involving terms like data structure, visualization, matplotlib.pyplot, and exploratory analysis suggest that students were also engaged with tasks involving data analysis and visual representation—key components in bioinformatics education. Additionally, the presence of terms like parameter, argument, and index indicates that the MCQs also addressed practical coding mechanics and syntax. Teams solved test problems using Python interactive notebooks, with access to the internet and consultation materials. The instructors provided guidance and feedback while encouraging that the teams were independent in solving the problems.

**Fig. 4:**
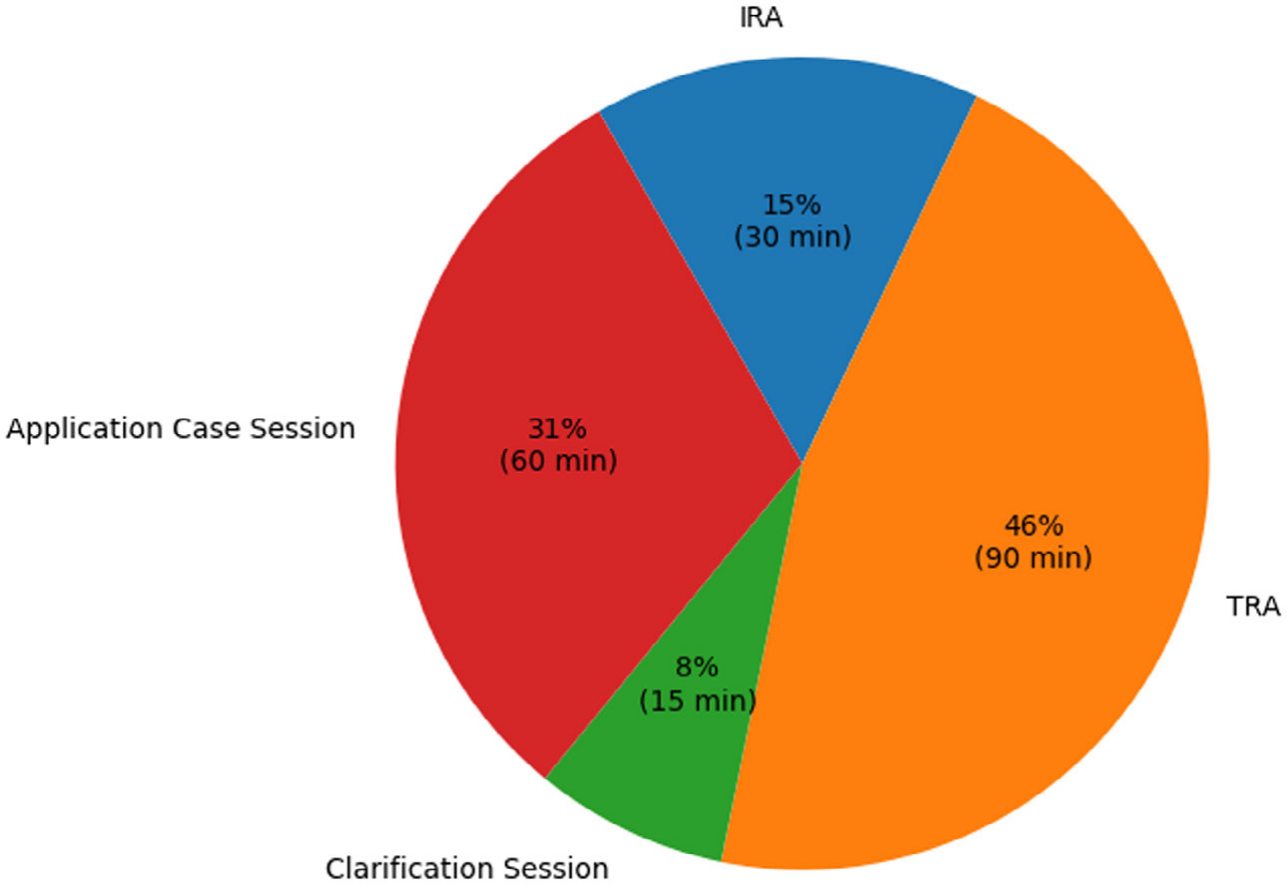
Overview of team‐based learning (TBL) Session Parts and Duration. This pie chart provides a visual representation of the distribution of time spent on different activities during a TBL session. The activities include the Individual readiness assurance test (IRA), the team readiness assurance test (TRA), a clarification session, and an application case session. The durations of these activities are represented as percentages of the total session time. The IRA, TRA, clarification session, and application case session account for 30 min (15.4%), 90 min (46.2%), 15 min (7.7%), and 60 min (30.8%) of the total session time, respectively. This figure illustrates the significant emphasis placed on the TRA in the TBL session, highlighting the importance of teamwork in this learning strategy.

The IRA scores were not formally evaluated to prevent disengagement, and students were aware that it was not being used for grading. The value of the IRA was explained to students as a tool for self‐identifying individual limitations in the topic before moving to teamwork. Students completed the IRA on paper and retained their individual tests and responses. They were encouraged to use their individual performance on the IRA for self‐reflection to identify areas of difficulty. The TRA and clarification session then provided a structured opportunity to address these difficulties. Teamwork was dynamic, with students actively discussing explanations and rationales for specific answers, including consulting material and testing code in the interactive notebooks. Informal feedback collected by tutors during teamwork and results from the clarification session indicated that all teams were able to reach the most correct answers in all 22 questions and justify their choices using accurate knowledge and correct rationales. This was followed by a 1‐h application case session where the teams were guided to explore the two additional notebooks and explore the real‐world applications (Fig. 4).

### Students' feedback

Following the session, an optional and anonymous survey was administered via Microsoft Forms to collect student ratings on teaching effectiveness. The survey consisted of seven items, which were chosen and slightly modified from the OIRA Item Bank50 and were rated on a 5‐point agreement scale (Supplementary Data S2).

**Fig. 5:**
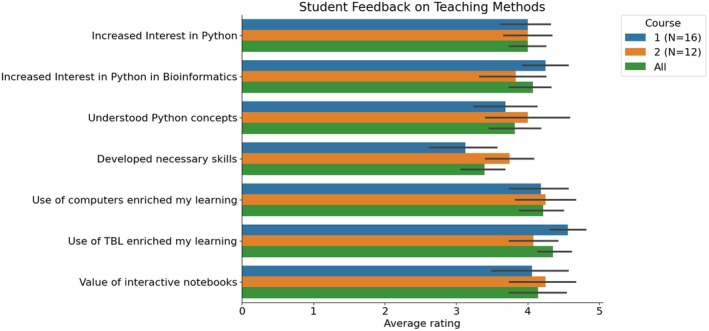
Student feedback on teaching methods. This horizontal bar chart represents the average ratings of student feedback on various teaching methods on each of the two courses and on the whole data. The y‐axis lists the seven statements that were rated, including ‘Increased Interest in Python’, ‘Increased Interest in Python in Bioinformatics’, ‘Understood Python concepts’, ‘Developed necessary skills’, ‘Use of computer enriched my learning’, ‘Use of TBL enriched my learning’, and ‘Value of interactive notebooks’. The *x*‐axis represents the average rating for each statement on a 5‐point agreement scale. The length of each bar corresponds to the average rating received for each statement, and the error bars indicate the 95% confidence intervals. The chart provides a visual representation of the students' perceptions and experiences during the session.

The average ratings across both courses (*N* = 28 students) for the statements indicated a high students' interest in Python (average rating of 4.0). There was also an increase in their interest in using Python in Bioinformatics analysis, as shown by an average rating of 4.1. The students reported gaining an understanding of major concepts related to Python, reflected by an average rating of 3.8. However, the development of skills necessary for researchers in this field received a moderate average rating of 3.4, suggesting an area for potential improvement. The use of the computer was found to enrich the students' learning experience in this class, as indicated by an average rating of 4.2. The use of team‐based learning (TBL) significantly enriched the students' learning experience in this class, as evidenced by a high average rating of 4.4. The students also found that interactive notebooks were a valuable part of the TBL session, as reflected by an average rating of 4.1 (Fig. 5 and Supplementary Data S2). While the use of github and Google Colaboratory was well received and facilitated broad access, some limitations related to institutional integration and long‐term service reliability should be considered (see Discussion and conclusions section below). We did not detect any significant differences between the answers on the two courses (*P* > 0.05, two‐sided Wilcoxon rank‐sum test).

Overall, these ratings provide a quantitative measure of the students' perceptions and experiences during the session. They indicate a positive shift in students' interest in Python, a good understanding of Python's major concepts, and a strong positive impact of TBL and the use of interactive notebooks on the learning experience. However, the rating for the development of necessary skills for researchers in the field was moderate, suggesting an area for potential improvement.

While the quantitative analysis of student feedback does not include a comparison with a control group or alternative teaching method, it offers useful insights into students' perceptions of this specific pedagogical intervention. As a descriptive case study, this analysis helps to contextualize student responses to the combined use of TBL and interactive Python notebooks within the course. We acknowledge the limitations of not having baseline or comparative data, which are often challenging to incorporate in real‐world academic settings where course structure and cohort size can constrain experimental design.

## Discussion and conclusions

The integration of team‐based learning (TBL) and cloud‐hosted interactive Python notebooks in bioinformatics education, as explored in this study, has demonstrated substantial potential in enhancing the learning experience of postgraduate biomedical students. This innovative pedagogical approach fostered an equitable environment that promotes active learning and critical thinking, aligning with previous research on TBL's effectiveness in health education [14, 15, 59, 60, 61, 62]. The implementation of TBL, known for its structured collaborative learning, facilitated active student participation and deeper engagement with the material through both individual and team efforts, which are crucial in mastering complex subjects like Python and its applications in bioinformatics [21].

The structure of TBL sessions, particularly the Individual and Team Readiness Assurance Tests (IRATs and TRATs), encouraged students to engage with the material at two levels: individually and collaboratively. This layered approach likely supported students' critical thinking and problem‐solving skills, key to understanding bioinformatics programming [18]. Based on the quality of team discussions during the TRA and the teams' ability to tackle the TRA questions, the TBL structure appeared to effectively incentivize pre‐class preparation, likely due to the accountability to team members. Consistent with findings from other studies using team‐based learning [16, 63, 64], the feedback we received from students highlights the positive impact of TBL in reinforcing key concepts in bioinformatics and promoting interactive learning. Future work could more directly evaluate the role and effectiveness of supplementary post‐class materials, such as the self‐study interactive notebook we provided here, in supporting skill retention and independent learning.

Unlike platforms that offer highly structured, auto‐graded coding exercises (e.g. DataCamp [65], CodeRunner [66]), our approach emphasized guided exploration within a TBL framework. This design was intentionally chosen to suit the goals of an intensive introductory session, where the focus was on developing conceptual understanding, promoting peer discussion, and familiarizing students with relevant computational tools. While auto‐graded platforms can support skill automation and detailed feedback, our method prioritized collaborative learning and foundational exposure within a limited timeframe.

We acknowledge that the structure of our TBL sessions differed from standard TBL delivery in several ways, reflecting adaptations made to address the specific needs of novice postgraduate students learning programming and bioinformatics. The extended duration allocated to the IRA and TRA was a deliberate choice, allowing students to actively engage with the interactive notebooks during the TRA phase. This approach blended readiness assurance with initial application, enabling teams to explore concepts, test their understanding, and build confidence in real time. Regarding the application exercises, while we aimed to maintain core TBL principles—particularly by presenting significant problems and encouraging rich team discussion—we adapted elements of the ‘4 S's’ framework. Specifically, ‘specific choice’ and ‘simultaneous reporting’ were less strictly applied due to the complex and open‐ended nature of coding tasks. These modifications were made to support a more exploratory learning experience, which we believe is essential for developing programming fluency in our educational context.

The use of cloud‐hosted interactive Python notebooks, linked to Google Colaboratory and github, proved to be a significant enhancement to the classroom experience. This approach addressed many of the technical barriers that often impede live coding sessions, as noted in previous work on jupyter notebooks and cloud platforms [27, 28, 29]. By integrating these tools, we created a seamless learning environment that ensured equitable access to computational resources, irrespective of students' technical capabilities or hardware limitations. The increased interest in Python, as reflected in the feedback, can be attributed to the accessibility and user‐friendly nature of this setup, which mirrors similar successful applications in other contexts [2, 33].

While the use of github and Google Colaboratory offered significant advantages in terms of accessibility, ease of deployment, and minimizing technical setup for students, it is important to acknowledge potential limitations associated with relying on third‐party cloud services. These include the risk of changes in service availability or functionality, limited integration with institutional virtual learning environments (VLEs), and constraints posed by institutional policies regarding external platforms. Additionally, although our course did not involve the use of sensitive data, concerns around data privacy and compliance should be carefully considered when scaling or replicating this approach. Future implementations may benefit from hybrid models that balance the flexibility of cloud tools with institutional infrastructure.

While students reported a moderate level of confidence in their Python skills by the end of the TBL session, this outcome is not surprising. Learning a new programming language like Python necessitates continuous practice, and the relatively short duration of the TBL session limited students' opportunities to fully develop their proficiency. This session was designed as an intensive introduction within broader postgraduate modules that also cover a wide range of biomedical topics. As such, achieving greater programming proficiency would require more dedicated course time, extended hands‐on practice, and potentially the inclusion of follow‐up workshops. This aligns with student feedback highlighting the need for further development in computational and research‐related skills to support their academic and professional goals. This finding underscores the importance of providing ongoing learning resources and support to help students further enhance their skills, as highlighted in other educational models [60]. Future iterations of the courses could benefit from incorporating additional follow‐up practice time, potentially combining Team‐Based Learning (TBL) with other educational strategies. One area of growing interest is the integration of AI chatbots, such as chatgpt, to enhance programming education [67]. Students' discussions and informal feedback offered valuable insights into their perceptions of AI's capacity to assist in generating Python code. Many recognized the potential benefits of AI tools for improving engagement and skill development by providing real‐time assistance and feedback. However, they also emphasized that researchers must understand the code being used to maintain accountability and uphold scientific rigor. Concerns were raised that the ability to automatically generate fully functional code in seconds might inadvertently discourage learners from fully engaging with the material. This reflects a broader concern within educational contexts regarding the responsible and effective integration of AI‐driven tools. Further research is needed to explore how AI‐driven tools can be integrated into programming education in ways that promote active learning and deep understanding, rather than fostering over‐reliance on automated solutions.

Overall, the combination of TBL and cloud‐hosted interactive Python notebooks has proven to be an effective pedagogical strategy for introducing biomedical students to Python and bioinformatics. This approach not only increased student engagement but also enhanced their perceived understanding of Python and its applications in bioinformatics, supporting it as a viable teaching method for similar courses. This study contributes to the growing body of literature on the benefits of TBL as a learning tool [14, 15, 59, 60, 61, 62]. Future research should focus on expanding this approach to further support skill acquisition and evaluate the impact of TBL on knowledge retention in this field.

While this study was conducted with a relatively small cohort of Master's‐level students (*N* = 28 in total), the pedagogical approach combining Team‐Based Learning (TBL) and cloud‐hosted interactive notebooks holds potential for broader application. The scalability of cloud‐based notebooks makes them well‐suited for larger classes by minimizing technical barriers and ensuring consistent access to computational resources. However, successful implementation of TBL at scale would require adequate teaching assistant (TA) support to facilitate team discussions, manage group dynamics, and provide timely feedback. With thoughtful planning and institutional support, this blended model could be adapted effectively for larger cohorts while maintaining its emphasis on active, collaborative learning.

This study is presented as a descriptive case study of an innovative approach to teaching Python in bioinformatics, focusing on implementation and student experience rather than comparative effectiveness. We acknowledge that the absence of a control or pre‐intervention measurement limits the generalizability of the quantitative feedback data. Nevertheless, in the context of real‐world teaching constraints, the findings offer valuable insights into how students engage with and perceive the use of Team‐Based Learning and cloud‐hosted notebooks. Future research could build on this work by incorporating longitudinal or comparative designs to further evaluate learning outcomes across instructional models.

This study highlights the success of combining Team‐Based Learning (TBL) with cloud‐hosted interactive Python notebooks to enhance bioinformatics education for postgraduate biomedical students. The approach promoted active learning, critical thinking, and equal access to resources, while also addressing common technical barriers. Students reported increased engagement, improved understanding of Python, and a greater interest in its application to bioinformatics. Although further practice was deemed necessary, the overall positive response supports the effectiveness of this strategy. The study offers a valuable model for educators aiming to implement innovative, collaborative methods in computational education.

## Conflict of interest

The authors declare no conflicts of interest.

## Author contributions

NSO conceptualized and designed the study. NSO and LDG implemented the teaching strategy, taught the courses, performed the analysis, and contributed to writing and editing the manuscript.

## Supporting information


**Data S1.** 22‐question individual readiness assurance test on python fundamentals.

**Data S2.** Students' survey results.

## Data Availability

All the data necessary to reproduce the analysis presented here is available in the supplementary materials. Course materials to reproduce our implementation in the classroom are available online at https://github.com/Leo-GG/Innovative-Pedagogy-in-Bioinformatics.
