# Supplementary material for: Teaching Python with team‐based learning: using cloud‐based notebooks for interactive coding education
Source: FEBS Open Bio. 2025 Aug 11;15(12):2054–66. doi: 10.1002/2211-5463.70097 (PMC12667212; doi:10.1002/2211-5463.70097)
Supplement: Supplementary file 1 — Data S1. 22‐question individual readiness assurance test on python fundamentals. [file FEB4-15-2054-s002.docx]

# **Python fundamentals for Bioinformatics # solved version**

**1. What is a variable in Python?**

A. A built-in function to output text or variables to the console.

B. A container used to store data, which is given a unique name.

C. A sequence of characters enclosed in single quotes, double quotes, or triple quotes.

D. A symbol that is ignored by the Python interpreter.

**2. Which of the following statements about the print function in Python is true?**

A. It is used to store data.

B. It is used to output text or variables to the console.

C. It is a sequence of characters enclosed in single quotes, double quotes, or triple quotes.

D. It is a symbol that is ignored by the Python interpreter.

**3. Which of the following statements about basic arithmetic operations in Python is true?**

A. Python does not support the use of parentheses to control the order of operations.

B. Basic arithmetic operations can only be performed on integers, not floats.

C. Python supports shorthand operators such as +=, -=, *=, and /=.

D. Basic arithmetic operations are not widely used in various programming fields.

+=: This operator adds the right operand to the left operand and assigns the result to the left operand. For example, a += b is equivalent to a = a + b.

-=: This operator subtracts the right operand from the left operand and assigns the result to the left operand. For example, a -= b is equivalent to a = a – b.

*=: This operator multiplies the right operand with the left operand and assigns the result to the left operand. For example, a *= b is equivalent to a = a * b.

/=: This operator divides the left operand by the right operand and assigns the result to the left operand. For example, a /= b is equivalent to a = a / b.

**4. Which of the following statements about conditional statements in Python is true?**

A. Conditional statements cannot control the flow of a program based on certain conditions.

B. Conditional statements do not allow you to check if a certain condition is true or false.

C. The most common conditional statements are the if-elif-else statements.

D. Conditional statements are not a fundamental concept in programming.

**5. Consider the following Python code:**


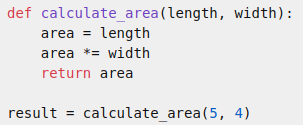


What does this code do?

A. The code defines a function named calculate_area that takes two parameters, length and width. It calculates the area by multiplying length and width, and returns the result. The function is then called with arguments 5 and 4, and the returned value is stored in the variable result.

B. The code defines a function named calculate_area that takes three parameters, length, width, and area, and returns the result. The function is then called, and the returned value, which is the product of 5 and 4, is stored in the variable result.

C. The code defines a function named calculate_area that takes two parameters, length and width. It initializes a variable area with the value of length, then multiplies area by width. The function then returns area. The function is called with arguments 5 and 4, and the returned value is stored in the variable result.

D. The code defines a function named calculate_area that takes two parameters, length and width. It initializes a variable area with the value of width, then multiplies area by length. The function then returns area. The function is called with arguments 5 and 4, and the returned value is stored in the variable result.

**6. Consider the following Python code:**


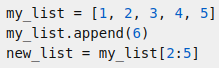


What is the value of new_list after executing this code?

A. [1, 2, 3]

B. [3, 4, 5]

C. [2, 3, 4]

D. [3, 4, 5, 6]

**7. In Python, if you have a list called my_list with 5 elements, what is the index of the first and last element?**

A. First element is at index 1 and last element is at index 5.

B. First element is at index 0 and last element is at index 4.

C. First element is at index 1 and last element is at index 4.

D. First element is at index 0 and last element is at index 5.

**8. Consider the following Python code:**


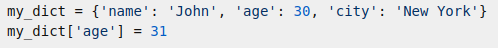


What is the value of my_dict after executing this code?

A. {'name': 'John', 'age': 30, 'city': 'New York'}

B. {'name': 'John', 'age': 31, 'city': 'New York'}

C. {'name': 'John', 'age': 30}

D. {'name': 'John', 'city': 'New York'}

**9. Consider the following Python code:**


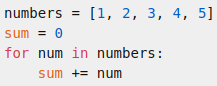


What is the value of sum after executing this code?

A. 0

B. 1

C. 15

D. 5

**10. What is a module in Python?**

A. A collection of code that can be imported and used in other code.

B. A data structure that allows you to store and organize collections of items.

C. A built-in function in Python that is used to output text or variables to the console.

D. A sequence of characters enclosed in single quotes, double quotes, or triple quotes.

**11. How do you import a module in Python?**

A. By using the module keyword followed by the name of the module.

B. By using the import keyword followed by the name of the module.

C. By using the load keyword followed by the name of the module.

D. By using the use keyword followed by the name of the module.

**12. Consider the following Python code:**


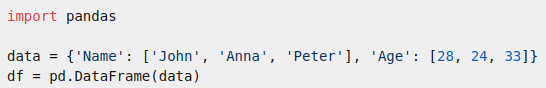


This code results in an error. What is the probable reason for the error?

A. The pandas library is not installed.

B. The pandas library is not correctly imported.

C. The DataFrame function is not correctly called.

D. The alias for pandas is not defined.

**13. What does the Pandas library in Python provide?**

A. Functions for creating high-quality images of molecules and visualizing protein structures.

B. Functions for reading, manipulating and writing data in a variety of formats.

C. Functions for analyzing single-cell gene expression data.

D. Functions for exploratory analysis of large-scale genetic variation data.

**14. What is the Seaborn library in Python used for?**

A. It is used for drawing attractive and informative statistical graphics.

B. It is used for analyzing single-cell gene expression data.

C. It is used for creating high-quality images of molecules and visualizing protein structures.

D. It is used for exploratory analysis of large-scale genetic variation data.

**15. What is the purpose of the Scanpy library in Python?**

A. It provides a high-level interface for drawing attractive and informative statistical graphics specifically for single-cell gene expression data.

B. It provides various bioinformatics tools for single-cell gene expression data, such as protein and DNA sequence analysis, molecular docking, and pharmacological prediction.

C. It includes preprocessing, visualization, clustering, trajectory inference, and differential expression testing for analyzing single-cell gene expression data.

D. It provides data structures and functions needed to manipulate single-cell gene expression data, including functions for reading and writing data in a variety of formats.

**16. What does the Biopython library in Python offer?**

A. It offers tools for sequence analysis, structure analysis, and biological data parsing.

B. It offers a scalable toolkit for analyzing single-cell gene expression data.

C. It offers a high-level interface for drawing attractive and informative statistical graphics.

D. It offers data structures and functions needed to manipulate structured data, including functions for reading and writing data in a variety of formats.

**17. What is the PyMOL library in Python used for?**

A. It is used for creating high-quality images of molecules and visualizing protein structures.

B. It is used for drawing attractive and informative statistical graphics.

C. It is used for analyzing single-cell gene expression data.

D. It is used for exploratory analysis of large-scale genetic variation data.

**18. What does the scikit-learn library in Python provide?**

A. It provides a variety of tools for classification, regression, clustering and feature selection.

B. It provides a high-level interface for drawing attractive and informative statistical graphics.

C. It provides tools for sequence analysis, structure analysis, and biological data parsing.

D. It provides data structures and functions needed to manipulate structured data, including functions for reading and writing data in a variety of formats.

**19. Consider the following Python code:**


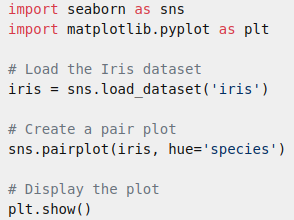


What does this code do?

A. Imports seaborn and matplotlib.pyplot, but does not load the Iris dataset, create any plot, or display anything.

B. Imports seaborn and matplotlib.pyplot, loads the Iris dataset, but does not create a pair plot or display anything.

C. Imports seaborn and matplotlib.pyplot, creates a pair plot of the Iris dataset, but does not use ‘species’ for coloring or display the plot.

D. Imports seaborn and matplotlib.pyplot, loads the Iris dataset, creates a pair plot colored by species, and displays the plot.

**20. Consider the following Python error message:**

ModuleNotFoundError: No module named 'malariagen_data'

What is the most likely reason for this error?

A. The malariagen_data module is a built-in Python module and does not need to be imported.

B. The malariagen_data module name is misspelled.

C. The malariagen_data module is not installed in the current Python environment.

D. The malariagen_data module is imported before it is defined in the code.

**21. Consider the following Python error message:**

TypeError: unsupported operand type(s) for +: 'int' and 'str'

What is the most likely reason for this error?

A. The code is trying to perform an addition operation and one of the operands is missing.

B. The code is trying to perform an addition operation and none of the operands is a number.

C. The code is trying to add two variables but one is a number and the other a string.

D. The code is trying to append two strings using the + operator.

**22. Consider the following Python error message:**

KeyError: 'NM_001256789'

What is the most likely reason for this error?

A. The gene identifier ‘NM_001256789’ does not exist in the NCBI database.

B. The gene identifier ‘NM_001256789’ is not present in the local dictionary.

C. The gene identifier ‘NM_001256789’ is misspelled or incorrectly formatted.

D. The gene identifier ‘NM_001256789’ is not a valid key for a Python dictionary.
